# Stent encrustation or fragmentation? A case report of post stent removal encrustation in postpartum woman and literature review

**DOI:** 10.1186/s12884-021-04262-x

**Published:** 2021-11-23

**Authors:** Zhaohua Ye, Qiwu Mi, Renzhao Huang

**Affiliations:** Department of Urology, Dongguan People’s Hospital, Dongguan, 523000 China

**Keywords:** Ureteral stent, Encrustation, Pregnant

## Abstract

**Background:**

Ureteral stents are commonly used in the field of urology to relieve ureteral obstruction. However, complications relating to ureteral stent use, such as encrustation continue to occur, especially with prolonged indwell time.

**Case presentation:**

Here we present a 37-year-old postpartum woman with a foreign body in her bladder after removing a ureteral stent 1 month before. She insisted that the foreign body was the fragment of stent and asked for medical malpractice indemnity payments while the surgeon of her insisted that the stent was intact during the procedure. Finally, the foreign body was confirmed as an encrustation by cystoscopy and the patient received 10,000 yuan ($ 1500) as indemnity payments after encrustation removal.

**Conclusion:**

In the absence of guidelines, stent indwelling time vary with centers’ habits, stent materials and patient’s education. Early detection of stent encrustation and timely removal of the encrusted stent are still the best way to avoid stent retention. Violent stent removal is of danger and not recommended in any case.

**Supplementary Information:**

The online version contains supplementary material available at 10.1186/s12884-021-04262-x.

## Background

Ureteral stents are commonly used in Urology to relieve ureteral obstruction [1, 2] and upper urinary calculus is a common cause of ureteral obstruction, especially in pregnant women [3]. Ureteral stenting is an effective surgical intervention when conservative treatments fail. However, indwelling ureteral stent can cause adverse effects or complications, including encrustation, especially with prolonged indwell time [4, 5]. There is still no optimal schedule for stent replacement. Frequent stent replacements might increase complications and economic burden [6]. Nowadays, although there are various methods to reduce encrustation formation [7–10], most of them have not been proven to be effective and safe in pregnant women and encrusted stent is particularly difficult to deal with in such population. Diagnostic imaging is the best way to detect stent encrustation [11, 12]. Standard KUB X-ray or CT scans always be the first choice for their satisfactory sensitivity and specificity, but the ionizing radiation exposure limits their use in pregnant women. Beside damage of the ureter, stent fragmentation, removing an encrusted stent forcibly may also cause the encrustation remain in the urinary tract and lead to corresponding complications. Early detection of stent encrustation and timely removal of the encrusted stent are the best way to avoid stent retention. In pregnant women, this requires a diagnostic tools with high sensitivity as well as safety.

## Case presentation

**Fig. 1:**
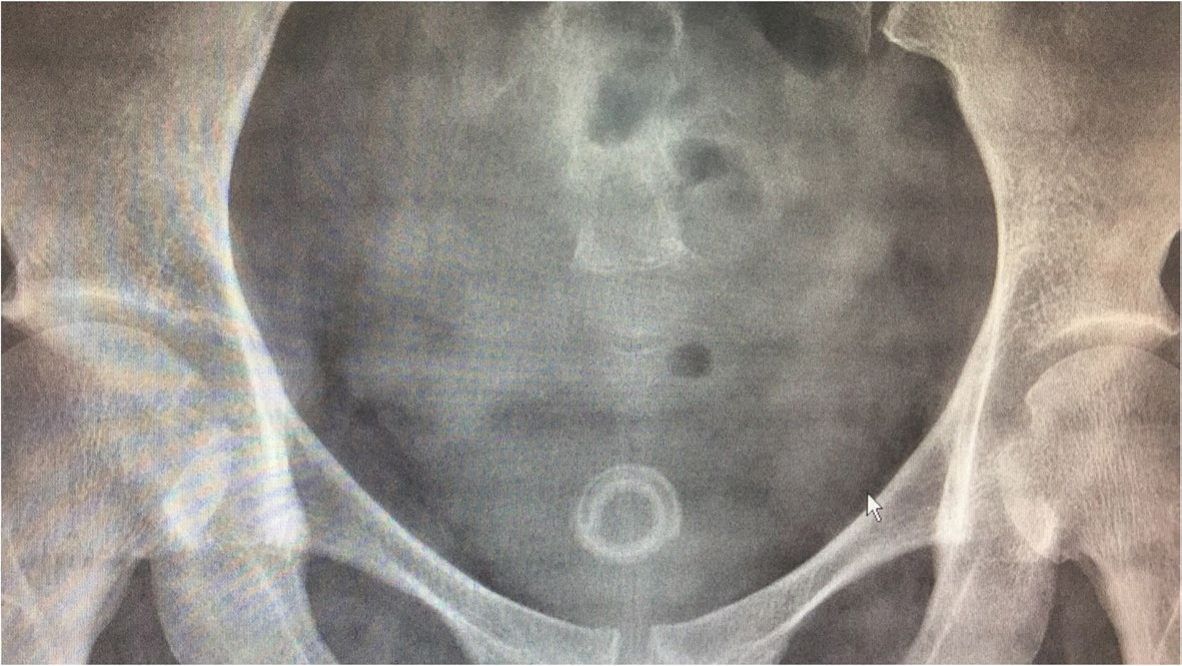
Plain kidney–ureter–bladder (KUB) radiograph demonstrated a high density foreign body in bladder

**Fig. 2:**
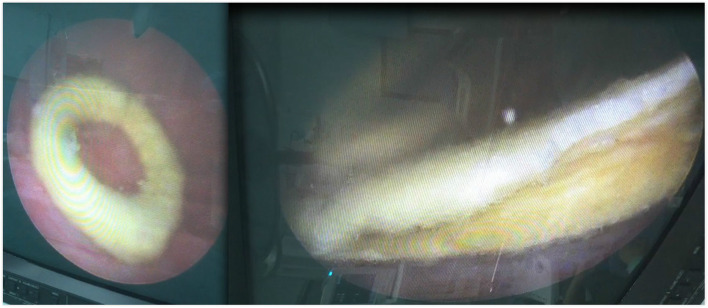
A donut-shape encrustation was found in the bladder with no stent fragment inside

**Fig. 3:**
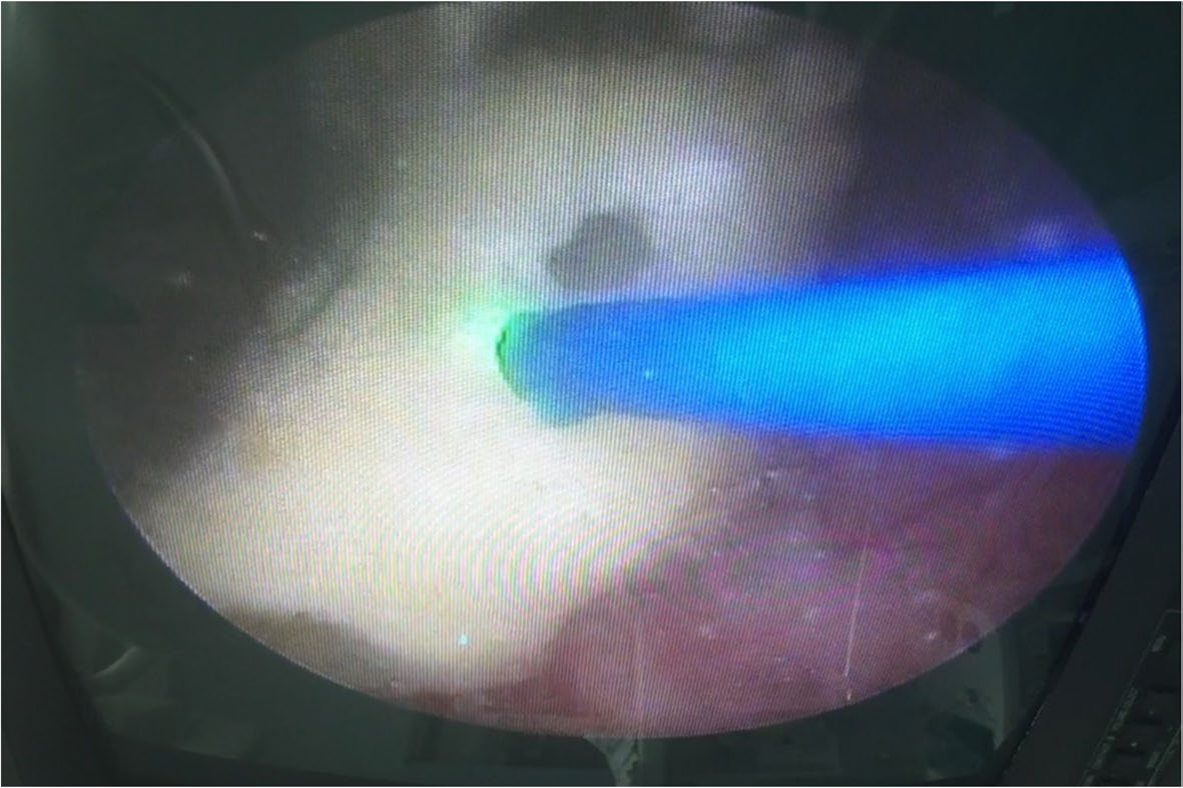
The encrustation was removed fragmented by holmium laser

A 37-year-old postpartum woman presented with a 1-month history of urgency, frequency and dysuria after removal of a 4 months double-J stent indwelt. She was initially treated as UTI but no improvement. Abdominal ultrasonography and KUB X-ray demonstrated hyperechoic and high density foreign body in bladder separately (Fig. 1). Considering the foreign body was the distal double-J stent fragment, she asked for medical malpractice indemnity payments. But the surgeon of her insisted that the stent was intact during the procedure. She underwent cystoscopy subsequently and a donut-shape encrustation was found in the bladder with no stent fragment inside (Fig. 2). The encrustation was removed fragmented by holmium laser (Fig. 3). The composition of the encrustation was a combination of brushite and weddellite. The patient fully recovered upon 1-month follow-up and she received 10,000 yuan ($ 1500) as indemnity payments finally.

## Discussion and conclusion

Since Zimsking and associates described the use of a ureteral stent to relieve ureteral obstruction in 1967, [13] ureteral stents had become the indispensable instruments in several urological procedures, [1, 2] particularly in those with obstruction due to urinary calculi, urinary stenosis, urogynecological tumors. Failure of conservative management is one of the indications for surgical intervention of urinary calculi in pregnancy and ureteral stent indwelling is the most frequent used techniques to all urologists because it can be performed under local anesthesia and ultrasound guidance without the risk of ionizing radiation [3]. However, serious complications such as encrustation, stone formation, infections, migration and fragmentation can be observed when the indwelling time is too long [4, 5].

In our case, the patient underwent stent (Cook Medical’s Black Silicone®, F7) indwelling after failure of conservative treatment and the whole indwelling time was over 4 months. Abdominal ultrasound was performed before stent removal but it was unable to detect the encrustation on the stent. The patient reported extreme painful during stent removal and severe hematuria after it. We believe that it is related to the encrustation of the distal coil, which prevented the distal coil from uncoiling and becoming stuck by the bladder neck. The reason for the surgeon did not notice the encrustation was that he only observed and clamped that part of the stent near the ureteral orifice without observing the distal coil. Although he did feel resistance when he drew the stent out, the intact of the stent mistaken him that everything was normal so he did not recheck the bladder.

Beside prolonged indwelling time, the risk factors of encrustation including stent materials, stent caliber, UTI, previous or concurrent stone disease, poor compliance, chronic renal failure, metabolic abnormalities, congenital renal anomalies and obstruction of the bladder outlet [3, 14–16]. Whether pregnancy is another risk factor to encrustation remains controversial. The increase of multiple lithogenic constituents of the urine during pregnant such as calcium, oxalate, uric acid and sodium is a trend towards an increased risk of encrustation. On the other hand, a similar increase in the excretion of urinary stone inhibitors including citrate, glycosaminoglycans, nephrocalcin, magnesium, uromodulin and thiosulfate, all which inhibit crystal growth and aggregation, may decrease encrustation formation [3, 17–19]. In our case, we consider the most important factor of encrustation was the long indwelling time. El-Faqih showed that the rate of complications was up to 76.3% when the stent maintained more than 12 weeks [20] and similar figures were observed by Kawahara [16]. Tunney MM similarly reported that 90% of ureteral stents had colonized pathogens and 55% had adherent biofilms [14]. MATTHEW F suggested that stent should be changed at least in 4 months and optimally every 2 months [21] while JOHN S. LAM suggest it should be shorter in those patients with risk factors that predispose them to developing encrustations [22]. There is no consensus on the ideal stent indwelling time for many urological procedures, but the temporal risk of encrustation is clear. Generally speaking, shorten the ureteral stent indwelling time is reasonable for most patients, but frequent stent exchange may also increase the risk of retrograde infection and financial burden [6]. Although silicone stents show lower rate of biofilm formation and mineral deposition [23] and large caliber stents (≥ 7F) are significantly associated with a lower encrustation rate [16], in our case these did not seem to counteract the complications caused by long-term stent indwelling..

Using metallic stents may be one method to reduce stent encrustation formation [7] and with safety for pregnant women. Drug-coated or drug-eluting stents that inhibit bacterial adhesion or mineral deposition have been proved can prevent the encrustation process [7, 8]. Biodegradable ureteral stents may partly solve the problem of forgotten ureteral stents [9, 10], but they are not suitable for the patients need long-term stent indwelling and the safety for pregnant women is still unknown.

The most common sites of encrustation were at the distal and proximal coil. Sighinolfi found that the composition of encrustation at the proximal coil reflected the composition of stones in patients with previous or concurrent stone disease. On the other hand, encrustation at the distal coil was related to UTI and bladder outlet dysfunction [24]. But this is not consistent with the conclusion of Roupret who did not find differences in the composition of encrustation at each coil of a stent [25]. In our case, encrustation only performed at the distal coil. We consider it is related to the fact that UTI and bladder outlet obstruction are more common in pregnancy women which agree with the outcome of Sighinolfi’s research [26]. Therefore, careful attention should be paid to the distal coil to determine the presence of encrustation before removing the stent in pregnant or postpartum women.

Imaging evaluation before stent removal is the most important way to detect stent encrustation. Standard KUB X-ray or CT scans always be the first choice with satisfactory sensitivity and specificity. There are several grading systems exist to define the extent of pathology and predict surgical complexity for stent removal, such as Acosta-Miranda’s “Forgotten, Encrusted, Calcified (FECal) system” [11] and Arenas’ “KUB system” [12]. Most of them are rely on radiation imaging, but the ionizing radiation exposure limits their use in pregnant women, particularly during the first trimester [27]. In China, pregnant or postpartum women always show excessive fear of ionizing radiation although multiple national and international organizations suggest that less than 50mGy is the accepted safe cumulative dose, with no increased risk of pregnancy loss or fetal anomalies [28, 29]. Low-dose or ultra-low-dose CT protocols have been recommended by AUA as an appropriate imaging modality for pregnant women in the second or third trimester when initial ultrasound is non-diagnostic [30]. MRI can be used for diagnostic imaging of pregnant patients with suspected urinary calculi as a second-line option [31]. Although it is considered as a reliable way for urinary calculi evaluation, there is no relevant data about its use in detecting stent encrustation. Ultrasound remains the initial diagnostic option for pregnant women because of the safety and availability, but conventional sonographic monitoring cannot detect stent encrustation well. Roman A. Blaheta and associates described the use of sonographic twinkling artfacts (TA) can be used to monitor early crystalline deposits in implanted ureteral stent in patients at risk for tumor lysis syndrome [32]. It might be an available choice for pregnant women with long-term stent indwelling. Pegah N and his colleagues had developed Quartz Crystal Microbalance (QCM) technique to leads to a faster comparison of different substrates and chemistries for studying the prevention of encrustation [33]. It might also have utility and safety in prediction of stent encrustation formation in pregnant women.

JOHN S. LAM suggested stopping further proceeding to avoid damaging the ureter if the patient complains of pain or the stent does not move easily. They considered that a slight coating was capable of locking the distal coils together to prevent uncoiling. Forced stent removal can result in ureteral injury or even ureteral avulsion [22]. It is accepted that removing a severe encrusted ureteral stent could be a difficulty problem for most urologists. Although numerous articles showed tips and tricks for solving this problem, it needs to be addressed in more than one approach, including ESWL, ureteroscopy, percutaneous nephrolithotomy and open surgery [34–36] and any associated stone burden must be addressed during the operative session [22].

Due to various complications caused by long-term stent indwelling, surgical management with ureteroscopy is an accepted reasonable alternative for pregnant women nowadays. It is reported that ureteroscopy had been shown to be both feasible and safe during the second and third trimesters of pregnancy with similar stone-free rates to non-pregnant patients [34]. A recent meta-analysis has demonstrated no difference in the incidence of ureteric injury or UTI in pregnant patients compared with non-pregnant patients [6]. Moreover, the post-procedural ureteral stent indwelling time is much shorter than delaying of definitive stone management until after delivery. If there are any contraindications of ureteroscopy and long-term ureteral stent indwelling must be performed instead, a reasonable follow-up strategy should be developed.

Shorten the stent indwelling time is the most effective preventive strategies for stent encrustation. Developing novel diagnostic tools to improve the early encrustation detection rate and inventing novel drug eluting stents which can be safely used in pregnant or postpartum women might be the course for the future. Forced stent removal is not recommended in any case despite of the stent can be removed intact occasionally, but it is more likely to result in serious complications and medical malpractice. Careful planning and combination of various surgical approaches are essential to remove a severe encrusted stent safely.

## Supplementary Information

**Additional file 1.**

**Additional file 2.**

**Additional file 3.**

**Additional file 4.**

## Data Availability

For further details, the corresponding author can be contacted.

## Abbreviations

UTI: Urinary tract infection
KUB: Kidney–ureter–bladder
ESWL: Extracorporeal shock wave lithotripsy

## References

[1] Saha PK, Hossain MS, Ghosh KC (2018). Forgotten, encrusted ureteral stents: removal - multimodal Endourologic approach. Mymensingh Med J.

[2] Gunduz N, Aslan A, Inan I (2017). A severely encrusted forgotten double J ureteral catheter. Eurasian J Med.

[3] Denstedt JD, Razvi H (1992). Management of urinary calculi during pregnancy. J Urol.

[4] Yoshida T, Inoue T, Taguchi M (2019). Efficacy and safety of complete Intraureteral stent placement versus conventional stent placement in relieving ureteral stent related symptoms: A randomized, prospective, single blind, multicenter clinical trial. J Urol.

[5] Tomer N, Garden E, Small A, Palese M (2021). Ureteral stent encrustation: epidemiology, pathophysiology, Management and Current Technology. J Urol.

[6] Semins MJ, Matlaga BR (2013). Management of urolithiasis in pregnancy. Int J Womens Health.

[7] Watterson JD, Cadieux PA, Beiko DT (2003). Oxalate-degrading enzymes from Oxalobacter formigenes: a novel device coating to reduce urinary tract biomaterial-related encrustation. J Endourol.

[8] Al-Aown A, Kyriazis I, Kallidonis P (2010). Ureteral stents: new ideas, new designs. Ther Adv Urol.

[9] Lumiaho J, Heino A, Tunninen V (1999). New bioabsorbable polylactide ureteral stent in the treatment of ureteral lesions: an experimental study. J Endourol.

[10] Schlick RW, Planz K (1998). In vitro results with special plastics for biodegradable endoureteral stents. J Endourol.

[11] Acosta-Miranda AM, Milner J, Turk TM (2009). The FECal Double-J: a simplified approach in the management of encrusted and retained ureteral stents. J Endourol.

[12] Arenas JL, Shen JK, Keheila M (2016). Kidney, ureter, and bladder (KUB): A novel grading system for encrusted ureteral stents. Urology.

[13] Zimskind PD, Fetter TR, Wilkerson JL (1967). Clinical use of long-term indwelling silicone rubber ureteral splints inserted cystoscopically. J Urol.

[14] Tunney MM, Keane PF, Jones DS, Gorman SP (1996). Comparative assessment of ureteral stent biomaterial encrustation. Biomaterials.

[15] Singh I, Gupta NP, Hemal AK, Aron M, Seth A, Dogra PN (2001). Severely encrusted polyurethane ureteral stents: management and analysis of potential risk factors. Urology.

[16] Kawahara T, Ito H, Terao H, Yoshida M, Matsuzaki J (2012). Ureteral stent encrustation, incrustation, and coloring: morbidity related to indwelling times. J Endourol.

[17] Borboroglu PG, Kane CJ (2000). Current management of severely encrusted ureteral stents with a large associated stone burden. J Urol.

[18] Masselli G, Derme M, Laghi F (2013). Imaging of stone disease in pregnancy. Abdom Imaging.

[19] Ordon M, Dirk J, Slater J, Kroft J, Dixon S, Welk B (2020). Incidence, treatment, and implications of kidney stones during pregnancy: A matched population-based cohort study. J Endourol.

[20] SR e-F, Shamsuddin AB, Chakrabarti A (1991). Polyurethane internal ureteral stents in treatment of stone patients: morbidity related to indwelling times. J Urol.

[21] Bultitude MF, Tiptaft RC, Glass JM, Dasgupta P (2003). Management of encrusted ureteral stents impacted in upper tract. Urology.

[22] Lam JS, Gupta M. Tips and tricks for the management of retained ureteral stents. J Endourol. 2002;16(10):733–41. 10.1089/08927790260472881 12542876

[23] Bouzidi H, Traxer O, Doré B (2008). Caractéristiques des incrustations des endoprothèses urétérales chez les patients lithiasiques [Characteristics of encrustation of ureteric stents in patients with urinary stones]. Prog Urol.

[24] Sighinolfi MC, Sighinolfi GP, Galli E (2015). Chemical and mineralogical analysis of ureteral stent encrustation and associated risk factors. Urology.

[25] Rouprêt M, Daudon M, Hupertan V, Gattegno B, Thibault P, Traxer O (2005). Can ureteral stent encrustation analysis predict urinary stone composition?. Urology.

[26] Kalinderi K, Delkos D, Kalinderis M, Athanasiadis A, Kalogiannidis I (2018). Urinary tract infection during pregnancy: current concepts on a common multifaceted problem. J Obstet Gynaecol.

[27] Brent RL, Mettler FA (2004). Pregnancy policy. AJR Am J Roentgenol.

[28] Anon: Committee Opinion No. 723: Guidelines for Diagnostic Imaging During Pregnancy and Lactation: Correction. Obstet Gynecol. 2018;132:786–6. 10.1097/AOG.0000000000002858 30134410

[29] Streffer C, Shore R, Konermann G (2003). Biological effects after prenatal irradiation (embryo and fetus). A report of the international commission on radiological protection. Ann ICRP.

[30] Fulgham PF, Assimos DG, Pearle MS, Preminger GM (2013). Clinical effectiveness protocols for imaging in the management of ureteral calculous disease: AUA technology assessment. J Urol.

[31] White WM, Johnson EB, Zite NB, et al. Predictive value of current imaging modalities for the detection of urolithiasis during pregnancy: a multicenter, longitudinal study. J Urol. 2013;189(3):931–4. 10.1016/j.juro.2012.09.076 23017526

[32] Blaheta RA, Oertl A, Freisleben HJ (2017). Detection of early DJ-stent encrustation by sonographic twinkling-artifacts - a pilot study. Cent European J Urol.

[33] Abadian PN, Buch PJ, Goluch ED, Li J, Zhang Z (2018). Real-time monitoring of urinary encrustation using a quartz crystal microbalance. Anal Chem.

[34] Duty B, Okhunov Z, Okeke Z, Smith A (2012). Medical malpractice in endourology: analysis of closed cases from the state of New York. J Urol.

[35] López-Huertas HL, Polcari AJ, Hugen CM, Farooq AV, Turk TM (2010). A novel technique for the removal of minimally encrusted ureteral stents. J Endourol.

[36] Aravantinos E, Gravas S, Karatzas AD, Tzortzis V, Melekos M (2006). Forgotten, encrusted ureteral stents: a challenging problem with an endourologic solution. J Endourol.

