# Supplementary figures and images for: Stent encrustation or fragmentation? A case report of post stent removal encrustation in postpartum woman and literature review
Source: BMC Pregnancy Childbirth. 2021 Nov 23;21:789. doi: 10.1186/s12884-021-04262-x (PMC8609729; doi:10.1186/s12884-021-04262-x)

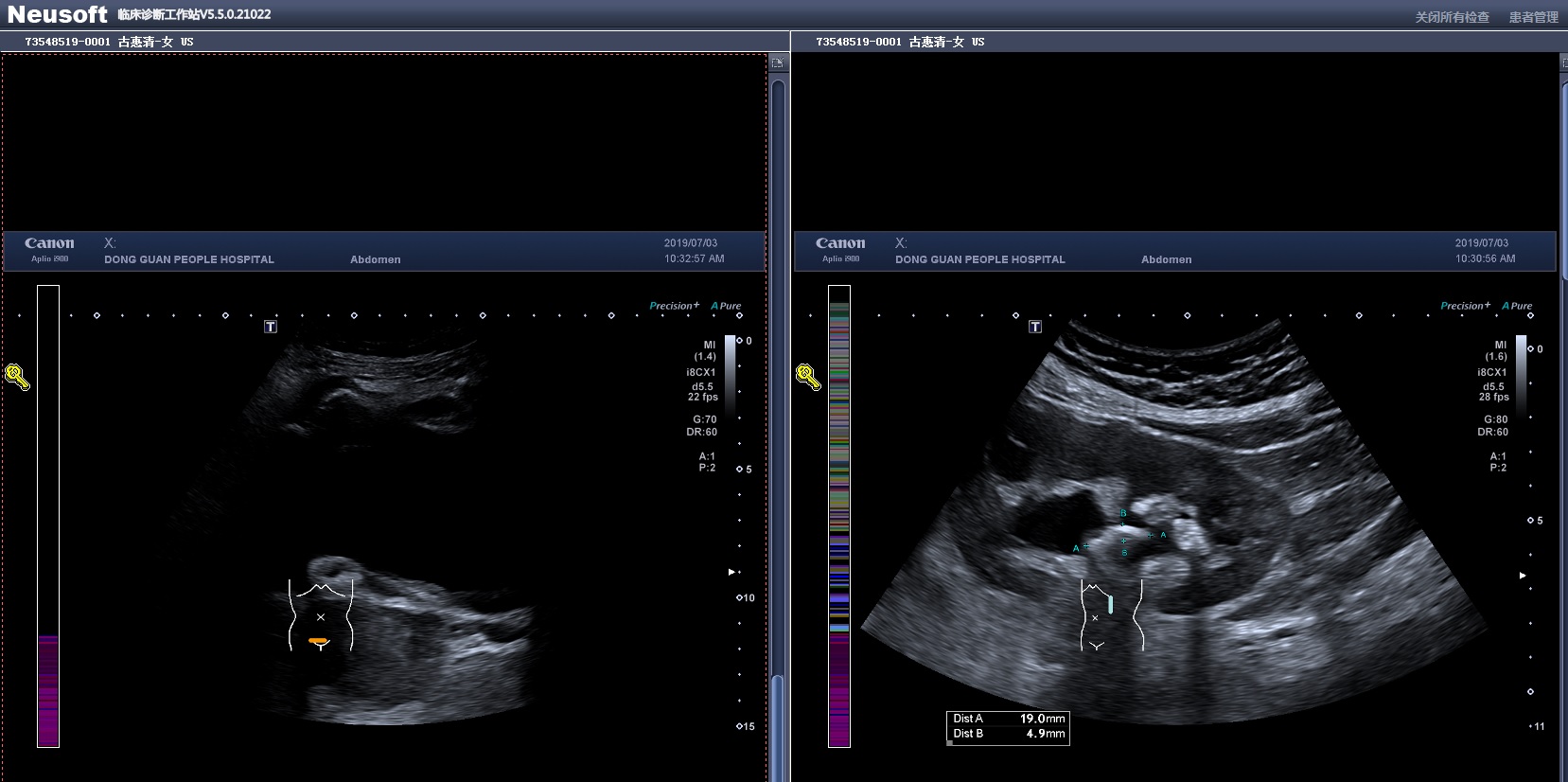

Supplement: Supplementary file 1 — Additional file 1. [file 12884_2021_4262_MOESM1_ESM.jpg]

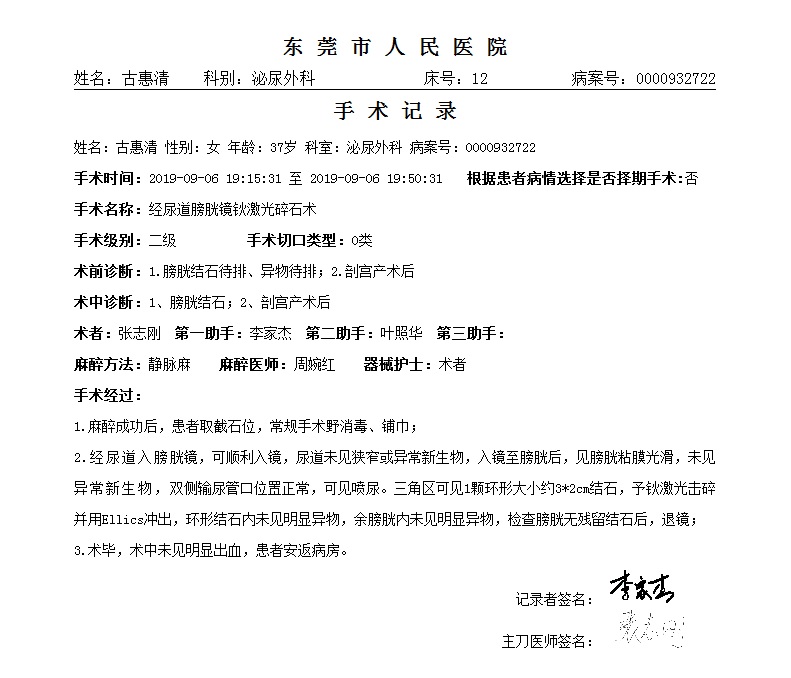

Supplement: Supplementary file 2 — Additional file 2. [file 12884_2021_4262_MOESM2_ESM.jpg]

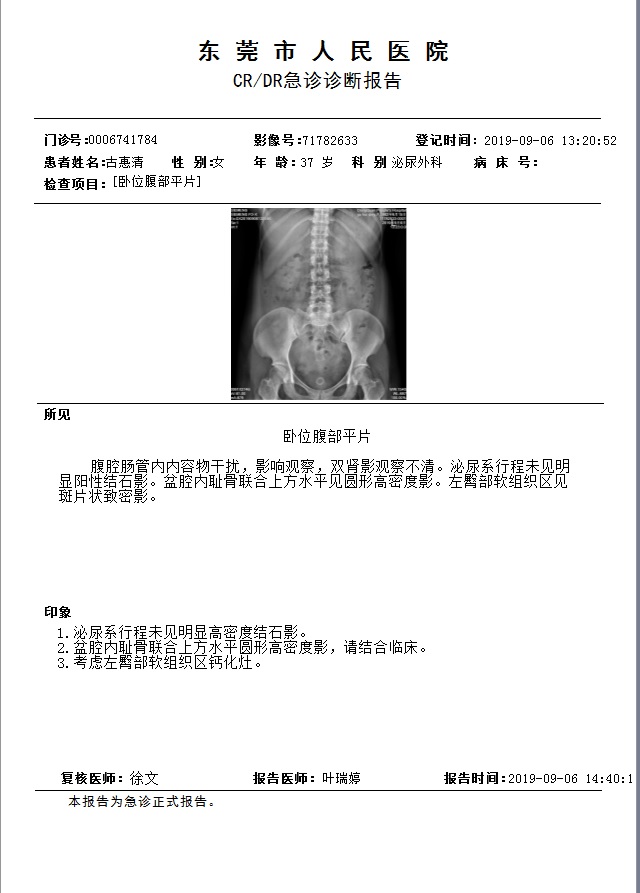

Supplement: Supplementary file 3 — Additional file 3. [file 12884_2021_4262_MOESM3_ESM.jpg]
